# Periodontal Ligament Mesenchymal Stromal Cells Increase Proliferation and Glycosaminoglycans Formation of Temporomandibular Joint Derived Fibrochondrocytes

**DOI:** 10.1155/2014/410167

**Published:** 2014-11-10

**Authors:** Jianli Zhang, Fujun Guo, Jianqiang Mi, Zhiye Zhang

**Affiliations:** ^1^Department of Stomatology, The First Affiliated Hospital of Henan, University of Science and Technology, 24 Jinghua Road, Jianxi District, Luoyang, Henan 471003, China; ^2^Department of Pathology, The First Affiliated Hospital of Henan University of Science and Technology, Luoyang 471003, China; ^3^Department of Oncology, The First Affiliated Hospital of Henan University of Science and Technology, Luoyang 471003, China

## Abstract

*Objectives*. Temporomandibular joint (TMJ) disorders are common disease in maxillofacial surgery. The aim of this study is to regenerate fibrocartilage with a mixture of TMJ fibrochondrocytes and periodontal ligament derived mesenchymal stem cells (PD-MSCs). *Materials and Methods*. Fibrochondrocytes and PD-MSC were cocultured (ratio 1 : 1) for 3 weeks. Histology and glycosaminoglycans (GAGs) assay were performed to examine the deposition of GAG. Green florescent protein (GFP) was used to track PD-MSC. Conditioned medium of PD-MSCs was collected to study the soluble factors. Gene expression of fibrochondrocytes cultured in conditioned medium was tested by quantitative PCR (qPCR). *Results*. Increased proliferation of TMJ-CH was observed in coculture pellets when compared to monoculture. Enhanced GAG production in cocultures was shown by histology and GAG quantification. Tracing of GFP revealed the fact that PD-MSC disappears after coculture with TMJ-CH for 3 weeks. In addition, conditioned medium of PD-MSC was also shown to increase the proliferation and GAG deposition of TMJ-CH. Meanwhile, results of qPCR demonstrated that conditioned medium enhanced the expression levels of matrix-related genes in TMJ-CH. *Conclusions*. Results from this study support the mechanism of MSC-chondrocyte interaction, in which MSCs act as secretor of soluble factors that stimulate proliferation and extracellular matrix deposition of chondrocytes.

## 1. Introduction

As the only joint in maxillofacial region, the temporomandibular joint (TMJ) is a bilateral synovial articulation between the mandible and temporal bone. In the middle of the mandibular condyle and the temporal bone lies the TMJ disc which is made of fibrocartilage. Disorders in TMJ cause pain and malfunction of jaw when chewing and talking. Degenerative diseases such as osteoarthritis are one of the frequently found disorders in TMJ [1]. Clinical options for treating these TMJ disorders are very limited since human cartilage tissue does not self-repair spontaneously [2]. Emerging techniques in stem cell biology and material science give hope to treat TMJ disorders with therapies based on tissue engineering approach [3, 4].

The TMJ disc mainly contains fibrocartilaginous tissue. Fibrochondrocytes resided in the tissue synthetized extracellular matrix (ECM) that predominantly contains collagen type I and collagen type II, as well as glycosaminoglycans (GAGs) [5, 6]. Two populations can be found in fibrochondrocytes: rounded shaped, chondrocyte-like cells and elongated, fibroblast-like cells [7]. Since the number of cells that can be obtained from small TMJ disc tissue biopsies is very limited, efforts are put in finding an alternative source of cells for regenerating fibrocartilage in TMJ disc [8, 9]. As a population of multipotent postnatal stem cells, the periodontal ligament derived mesenchymal stem cells (PD-MSCs) are easily accessible for oral surgeons [10]. They can be propagated* in vitro* to provide an excellent source of adult stem cells with biological properties similar to mesenchymal stem cells derived from other tissues like bone marrow and fat tissue [11]. Useful especially for dental tissue regeneration, PD-MSCs had been shown to differentiate into periodontal fibroblasts, osteoblasts, and cementoblasts [12]. Allogeneic transplantation had been reported to repair bone defects without immunological rejection, due to their immunosuppressive effects [13]. It has also been reported that PD-MSCs were useful in periodontitis [14]. For these reasons, a coculture system of MSCs and TMJ chondrocytes could be an attractive cell source for TMJ tissue engineering.

Recently, a group of papers emphasized the supportive effects of MSCs, instead of chondrogenic potential of MSCs in coculture systems of MSCs and chondrocytes. Using a xenogenic coculture model of human MSCs and bovine chondrocytes, Wu et al. showed that the beneficial effects of the coculture are largely due to increased chondrocyte proliferation and matrix formation induced by MSCs [15]. Furthermore, a significant decrease of MSCs in coculture pellets was observed, resulting in an almost homogeneous cartilage tissue in 3 weeks. Similar results were obtained by other research groups [16, 17]. These studies together demonstrated a new mechanism of cellular interaction between MSCs and chondrocytes: MSCs support chondrocyte proliferation and cartilage matrix deposition rather than actively undergo chondrogenic differentiation.

In this study, we investigated the supportive effects of PD-MSC on chondrocytes derived from TMJ disc. Cell proliferation, matrix production, and chondrogenic gene expressions were examined in TMJ chondrocytes after coculture with PD-MSC. These results could be important for developing tissue engineering approach that treats TMJ disorders.

## 2. Materials and Methods

### 2.1. Sample Collection and Cell Isolation

TMJ cartilage was isolated from the retrodiscal area of human adult TMJ discs of patients with temporomandibular disorders (TMD) who under arthroscopic examinations. Biopsies were chopped into small pieces of about 2 mm × 2 mm. Then, tissue pieces were digested overnight in DMEM containing 10% FBS, 1 mg/mL collagenase type II (Worthington, Lakewood, NJ). After digestion, cells were washed with PBS and seeded into tissue culture flasks in expansion medium (DMEM supplemented with 10% FBS, 1% penicillin/streptomycin, all from Sigma-Aldrich). Normal impacted third molars were obtained from patients at the Dental Clinic of Department of Stomatology, following approved guidelines set by Henan University of Science and Technology. Periodontal ligament tissue was gently separated from the surface of the root and then digested in DMEM containing 1 mg/mL collagenase type I (Worthington, Lakewood, NJ) overnight. PD-MSCs were washed with PBS and seeded into tissue culture flasks in DMEM supplemented with 10% fetal bovine serum (FBS), 1% penicillin/streptomycin. Samples from 3 donors of MSCs were pooled. TMJ fibrochondrocytes of six donors were collected individually for this study. All patients gave their written consent to participate in the study. This work was approved by the Research Ethics Committee of the Henan University of Science and Technology.

### 2.2. Labeling of PD-MSC with GFP Lentivirus

PD-MSCs at passage 1 were transduced with GFP lentivirus with puromycin resistant gene (Cyagen, Guangzhou, China). Two days after transduction, puromycin (1 *μ*M) was added to expansion medium. PD-MSC was then expanded in medium with puromycin for two weeks until coculture with TMJ chondrocytes.

### 2.3. Pellet Culture of PD-MSC and TMJ Chondrocytes

For the mixture group, 100 000 PD-MSCs and 100 000 TMJ chondrocytes (ratio = 1 : 1) were seeded in one well of a round bottom ultralow attachment 96-well plate (Corning, Lowell, MA) in expansion medium and centrifuged for 3 min at 2000 rpm. For PD-MSC or TMJ-CH groups, 200 000 corresponding cells were seeded in the same plate with the same medium. Medium was refreshed twice a week.

### 2.4. Histology

Cell pellets were processed with routine histological procedures. Briefly, cell pellets were fixed with 10% formalin, followed by embedding in Paraffin. Sections of 4 *μ*m were cut and stained for glycosaminoglycans (GAGs) with Alcian Blue combined with counterstaining of nuclear fast red to visualize nuclei.

### 2.5. Quantitative GAG and DNA Assay

Six pellets from each group were collected for GAG and DNA assay. For mixture and TMJ-CH groups, one pellet was collected from one donor of TMJ fibrochondrocytes; for MSC only, 6 pellets were made from a pool of 3 donors. Cell pellets were frozen overnight at −80°C, followed by digestion in 500 uL digestion buffer (1 mg/mL proteinase K in Tris/EDTA buffer (pH 7.6)) for more than 16 h at 56°C. GAG content was spectrophotometrically examined with 1,9-dimethylmethylene blue chloride (DMMB) staining in PBE buffer (14.2 g/L Na2HPO4 and 3.72 g/L Na2EDTA, pH 6.5) using a microplate reader (TECAN, Grodig, Austria) at an absorbance of 520 nm, using chondroitin sulfate as a standard. Total DNA was demined using a CyQuant DNA Kit (Molecular Probes, Eugene, OR) as representative of cell numbers for normalization of GAG.

### 2.6. DNA Isolation, RNA Isolation, and Quantitative PCR

Genomic DNA of pellets was extracted with the QIAamp DNA Mini Kit (Qiagen, Hilden, Germany). Total RNA of cell pellets was isolated with an RNeasy Mini Kit (Qiagen, Hilden, Germany). An iScript cDNA Synthesis kit (Bio-Rad, Hercules, CA) was used to reverse-transcribe one microgram of total RNA into cDNA. Real time PCR (qPCR) was performed on genomic DNA or cDNA samples by using the iQ SYBR Green Supermix (Bio-Rad, Hercules, CA). PCR reactions were carried out on MyiQ2 Two-Color Real-Time PCR Detection System (Bio-Rad, Hercules, CA). For each reaction, a melting curve was generated to test primer dimer formation and nonspecific priming. The primers sequences for chondrogenic genes were obtained from Primer Bank of Harvard University (http://pga.mgh.harvard.edu/primerbank/). Primers for GFP are as follows: forward (TGTTCCATGGCCAACACTTG) and reverse (ACGTGTCTTGTAGTTCCCGT). Relative expression was calculated using the double delta Ct method [18].

### 2.7. EdU Labeling, Staining, and Quantification

A Click-iT EdU Imaging Kit (Molecular Probes, Eugene, OR) was used to examine the proliferation of cells in pellets. EdU (5-ethynyl-2′-deoxyuridine) was added to the culture media at a concentration of 10 *μ*M, 48 h after seeding in 96-well plate. A cryotome (Leica, Germany) was used to make 10 *μ*M sections. EdU staining was performed with manufacturer's protocol. Nuclei were counterstained with Hoechst 33342. Then, fluorescent images were taken with a DMi 6000 B fluorescent microscope (Leica, Bensheim, Germany). Images were analyzed according to previously published method [15]. Briefly, we manually set a threshold to avoid artifacts. The number of green cells, red cells, green + red cells, and total cells was counted by running plug-ins written in macro language of ImageJ (available on request). Green cells accounted for MSCs; non-green cells were fibrochondrocytes; red cells were proliferating cells; green + red cells were proliferating MSCs; non-green + red cells were proliferating fibrochondrocytes. Values represent the mean ± standard deviation of at least 3 biological replicates.

### 2.8. Collection of Conditioned Medium

DMEM was incubated with 90% confluent PD-MSCs for 48 h to collect secreted factors made by MSC. Protein fraction of the medium was concentrated for about 100-fold using an Amicon Ultra-15 Centrifugal Filter Unites (Millipore, Billerica, MA) with a cut-off of 3000 daltons. The concentrated solute was supplemented with 10% FBS and antibiotics to make conditioned medium.

### 2.9. Statistical Analysis

Both one-way analysis of variance (ANOVA) and Student's test were used for statistical analysis. Method for individual experiment was indicated in figure legends.* P* values of <0.05 were considered as significant.

## 3. Results

### 3.1. PD-MSC Increases Proliferation and GAG Production of TMJ-CH

**Figure 1:**
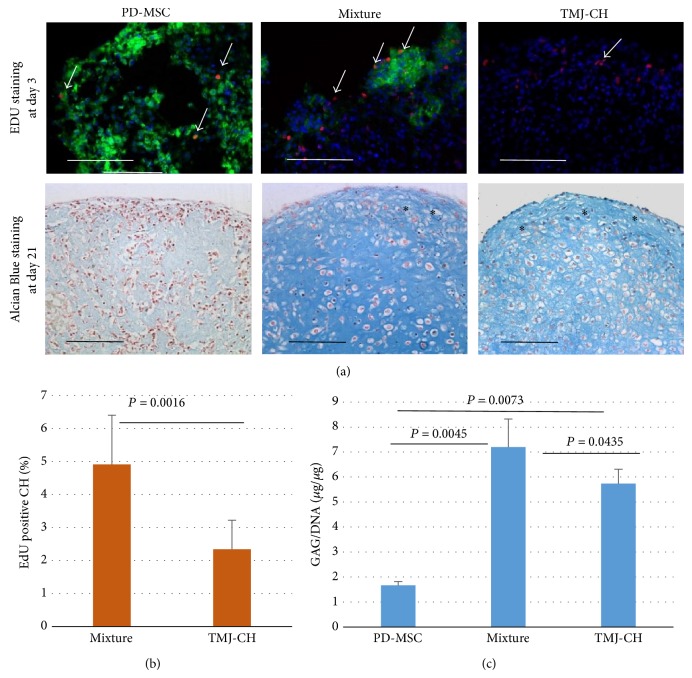
PD-MSCs increase proliferation and GAG formation of TMJ-chondrocytes. (a) EdU staining and Alcian Blue staining of monoculture and coculture pellets. At day 3 after aggregation, EdU staining was performed to detect proliferating chondrocytes. EdU positive cells are visualized by Alexa 594 (red), as indicated by white arrowhead. PD-MSCs are marked by GFP (green), and nuclei were counterstained with Hoechst 33342 (blue). At day 21 after aggregation, GAGs were stained by Alcian Blue. Fibrotic tissue was indicated by asterisk. Scale bar = 100 *μ*m. (b) Quantification of EdU positive chondrocytes. Quantification is based on the assumption that all non-green cells are TMJ chondrocytes. Data from 3 donor pairs were calculated to show mean ± standard deviation. Statistical significance was analyzed by Student's* t*-test. (c) GAG quantification. Quantitative GAG assay shows more GAGs in mixture group than in the other two groups (*n* = 6) at week 3 after aggregation. Error bar reflects standard deviation. *P* values were calculated with one-way ANOVA followed by Dunnett's test.

PD-MSCs were labeled with GFP with stable virtual transduction to enable long-term tracking in cocultures system. PD-MSCs were then mixed with chondrocytes derived from TMJ (TMJ-CH) at a ratio of 1 : 1 to make pellets. Pellets containing PD-MSC only or TMJ-CH only were used as controls. All pellets from three groups were cultured in expansion medium. At day 3, proliferation of chondrocytes was examined with EdU incorporation, since most proliferative activities happened in a few days after cell aggregation [15]. As shown in Figure 1(a) upper panel, EdU positive cells were mainly distributed on the surface of coculture pellets (mixture) and chondrocytes pellets (TMJ-CH), but more homogenously in MSC pellets (PD-MSC). Proliferating chondrocytes were quantified by counting EdU positive cells in the non-green area of the pellets. As shown in Figure 1(b), there was about 5% EdU positive chondrocytes in mixture group, while only ~2% chondrocyte in TMJ-CH group trying to replicate DNA. Differences are statistically significant. At week 3, Alcian Blue staining and GAG/DNA assay were performed to examine deposition of sulfate GAG. Blue staining was shown in mixture group and TMJ-CH group, which indicated the presence of GAG in the pellets (Figure 1(a) lower panel). Very few GAGs were observed in PD-MSCs group since no growth factors were present in the expansion medium. In general, cells in the positively stained areas showed chondrocyte morphology. Monoculture of TMJ-CH showed more fibers in matrix than coculture pellets. Quantification of GAGs confirmed our impression that mixture group contained more GAGs than TMJ-CH group. This indicated that PD-MSC increased the matrix formation of chondrocytes in cocultures. These data are in line with previously reported data.

### 3.2. PD-MSC Disappears after Coculture with TMJ-CH

**Figure 2:**
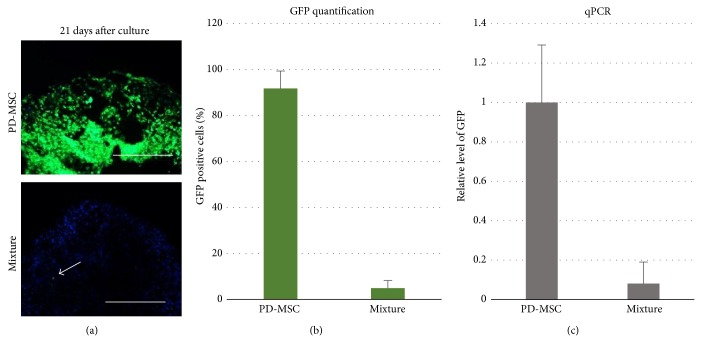
PD-MSCs disappear after coculture. (a)  Fluorescent images of pellets at 21 days after aggregation. Cryosections were made without fixation. Scale bar = 100 *μ*m. Arrowhead indicates remaining green cells in coculture pellets. (b) Fluorescent images were analyzed to quantify GFP positive cells. Data from 3 donor pairs were calculated to show mean ± standard deviation. (c) Quantitative PCR of GFP. GAPDH was amplified on genomic DNA to stand for cell numbers. GFP was amplified and normalized to GAPDH. PD-MSC was chosen as reference. Number in mixture group represents the relative amount of GFP compared to PD-MSC. Three donor pairs were analyzed.

To track PD-MSC after coculture, cryosections were made to examine GFP signaling in the pellets. As shown in Figures 2(a) and 2(b), barely few green cells can be seen in coculture pellet (below 5%) after 3 weeks culture, while in monoculture of PD-MSC majority of cells are GFP positive (more than 90%). To exclude the possibility that GFP labeled PD-MSCs become quiescent, genomic DNA of PD-MSC and mixture group was extracted to perform real time qPCR for GFP. GAPDH was also amplified for normalization. In line with image quantification (Figure 2(b)), pellets of mixture group contained only a small amount of GFP DNA (~8%) comparing to pellets of PD-MSC group.

### 3.3. Conditioned Medium of PD-MSCs Increases Proliferation and Matrix Formation of TMJ-CH

**Figure 3:**
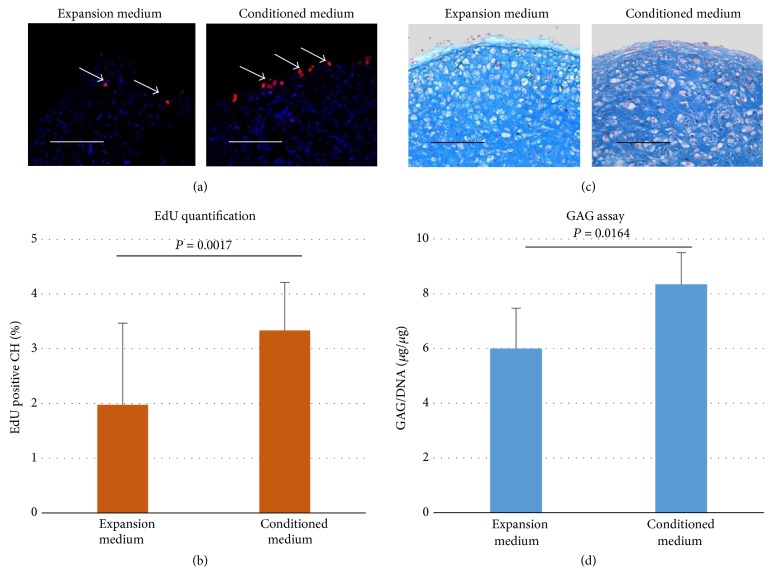
Conditioned medium increases proliferation and GAG deposition. (a)  EdU staining was performed at day 3 after aggregation. Positively stained cells are visualized by Alexa 594 (red), as indicated by white arrowhead. Nuclei were counterstained with Hoechst 33342 (blue). Scale bar = 100 *μ*m. (b)  EdU positive cells were quantified based on fluorescent images (*N* = 3). Data is shown as mean ± standard deviation. Statistical significance was analyzed by Student's* t*-test. (c)  Alcian Blue staining was carried out at day 21 after aggregation for GAGs. Scale bar = 100 *μ*m. (d)  GAG contents in pellets were assayed at day 21 after aggregation (*n* = 6). Error bar reflects standard deviation. *P* values were calculated with Student's* t*-test.

To verify if the supportive effects are mediated by secreted factors, conditioned medium of PD-MSCs was collected to culture TMJ-CH pellets. As shown in Figures 3(a) and 3(b), pellets of TMJ-CH cultured in conditioned medium for 3 days contained significantly more EdU positive cells than pellets cultured in expansion medium. Interestingly, the pattern of how EdU positive cells are distributed in the pellets is similar to that of coculture pellets, which indicates that the effect of PD-MSCs conditioned medium is close to PD-MSC coculture. After 3-week culture in conditioned medium or expansion medium, GAGs were measured and normalized to DNA. Pellets cultured in conditioned medium contained significantly more GAGs than those cultured in expansion medium (Figures 3(c) and 3(d)).

### 3.4. Conditioned Medium of PD-MSC Increases Expression of Chondrogenic Genes in TMJ-CH

**Figure 4:**
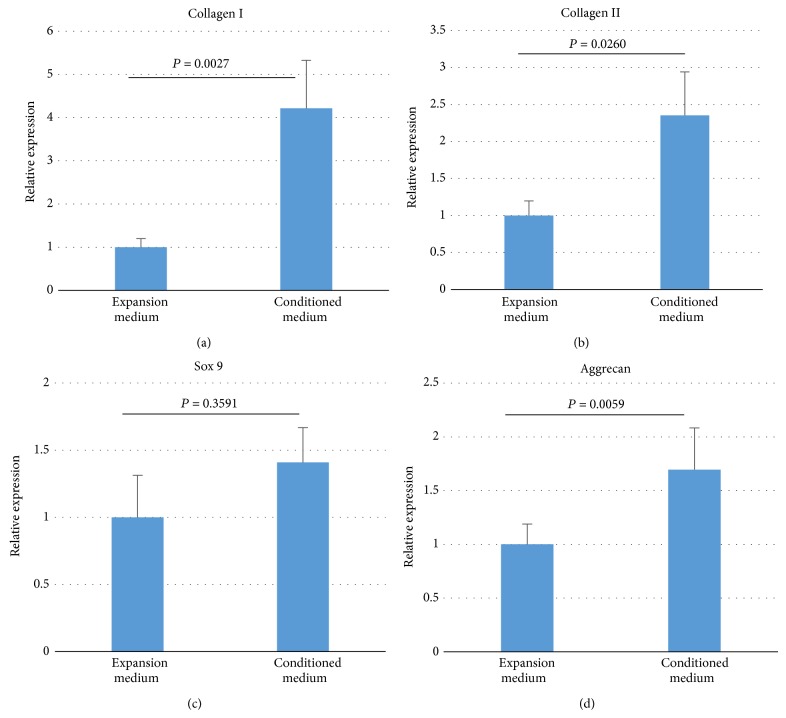
Conditioned medium increases chondrogenic gene expression. Real time qPCR for collagen type I (a), collagen type II (b), Sox 9 (c), and Aggrecan (d) was performed on RNA samples extracted from pellets cultured either in expansion medium or conditioned medium for 3 weeks (*N* = 4). Geometric average of GAPDH and *β*-actin was used for normalization. Expression level in pellets cultured in expansion medium was chosen as reference. *P* values were calculated with Student's* t*-test.

To explain how conditioned medium increases GAG production of TMJ derived chondrocytes, real time qPCR was performed to study the expression of chondrogenic genes in pellets cultured in either expansion medium or conditioned medium for 3 weeks (Figure 4). Three out of four genes we tested are significantly higher in pellets cultured in conditioned medium than those in expansion medium. These data suggested that the conditioned medium of PD-MSC increases cartilage matrix deposition by promoting the expression of chondrogenic genes.

## 4. Discussion

In this study, a coculture model of TMJ-CH and PD-MSC was used to study the supportive effects of MSCs on fibrochondrocytes. We demonstrated that proliferation and GAG deposition of TMJ-CH were increased in coculture pellets when compared to pellets of TMJ-CH only. Ratio of PD-MSC decreased after coculture as tracked by GFP. Finally, we showed that the supportive effects of MSC to fibrochondrocytes are mediated through soluble factors.

Since first reported in 1990s, mesenchymal stem cells (MSCs) have been considered as the most promising cell source for tissue engineering and regenerative medicine [19]. One of the most important features of MSC is their potential to differentiate into multiple lineages, no matter where they are isolated from [20, 21]. In recent years, supportive effects of MSCs have been proposed and drawn wide attention, as MSCs did not differentiate into tissue-specific cell type while still benefited tissue repair in many cases [22]. Like a coin with two sides, both differentiation potential and supportive effects may play important role in a broad spectrum of application in tissue engineering. However, which side is shown up when MSCs meet chondrocytes is still of debate. It has been reported that coculture of MSCs and nucleus pulposus cells in 3-dimensional environments induced chondrogenic gene expression in MSCs [23]. Conditioned medium of chondrocytes was also reported to induce osteochondrogenic differentiation of MSCs [24]. On the other hand, many groups also published papers in favor of MSCs' supportive effects [16, 25, 26]. Here we report that coculture of TMJ derived chondrocytes and PD-MSCs benefits cartilage formation and the effects are largely due to increased proliferation and GAG production of TMJ chondrocyte. We also concluded that PD-MSCs do not actively undergo differentiation into chondrocytes since very few portions of PD-MSCs survived after coculture.

Regarding the mechanism of how PD-MSCs support chondrocytes, our data suggested the effects are most likely mediated through soluble factors secreted by PD-MSCs. It has been reported that MSCs expressed and secreted significant amounts of basic fibroblast growth factor (bFGF), vascular endothelial growth factor A (VEGF-A), and interleukin 6 (IL-6) and interleukin 8 (IL-8) [27]. Conditioned medium of MSCs was reported to promote wound healing in a scratch model* in vitro* by enhancing migration and matrix deposition of dermal fibroblasts [28]. MSCs transplanted at injury sites of neural system could promote functional recovery of nerves by secreting trophic factors that induce survival and regeneration of host neurons [29]. di Bernardo et al. reported that placenta derived MSCs are source of paracrine factors that stimulate pulmonary morphogenesis in a fetal organ culture system [30]. One more specific example on cartilage regeneration is that fibroblast growth factor-1 (FGF-1) is reported to be produced by MSCs in coculture with articular chondrocytes, with stimulatory effects on chondrocytes proliferation and matrix formation [31]. Adding to all these examples, we showed in this study that secreted factors from MSC could support proliferation and matrix formation of fibrochondrocytes. We demonstrate that, to some extent, fibrochondrocytes isolated from TMJ discs can respond to trophic factors produced from MSCs, similar to their counterpart in articular cartilage.

Recently, several attempts have been made to regenerate fibrocartilaginous tissue with tissue engineering approaches [32–34]. Most of these studies emphasize on the optimization of scaffolds or growth factors used for fibrocartilage engineering. Very few papers focus on cell sources suitable for fibrocartilage regeneration [35]. Suggested by the result of the present study, a mixture of PD-MSC and TMJ derived fibrochondrocytes could be a good alternative source of cell for fibrocartilage engineering. As mentioned above, MSCs produce significant amount of TGF-*β*, FGF-1, and FGF-2 which may induce fibrogenic phenotype on articular chondrocytes [36]. This observation raises concerns when a mixture of MSCs and chondrocytes is used to regenerate hyaline cartilage, since it is possible that fibrocartilage may fill the injury site on articular surface with inferior mechanical properties [37]. Recent studies suggested that coimplantation of MSCs and articular chondrocytes may be better than chondrocyte only to repair cartilage defects referring to the number of chondrocytes needed for implantation [15, 16]. With the trophic effects of MSCs, less number of chondrocytes is needed to fill the defective sites. These studies mainly emphasized GAG formation and collagen II deposition though. No attention was paid for collagen I synthesis. Based on published data, it is not clear if coculture or coimplantation would increase fibrogenesis of articular chondrocytes. While this is still under debate, however, fibrogenesis may be an advantage for regeneration of fibrocartilage such as meniscus, intervertebral disc, and TMJ disc. For TMJ disc repair especially, PD-MSC could substitute for the insufficient number of chondrocytes isolated from TMJ. Growth factors secreted from PD-MSC may simulate the proliferation and matrix deposition of chondrocytes. Combined with proper scaffolds, TMJ discs could be regenerated with relatively small number of chondrocytes.

Taken together, our data demonstrated that, by secreting soluble factors, MSCs derived from periodontal ligament supported the proliferation and GAG production of fibrochondrocytes derived from TMJ. Results from this study may provide new cell source for developing tissue engineering based therapeutics for treating TMJ disorders.

## References

[1] Kalladka M., Quek S., Heir G., Eliav E., Mupparapu M., Viswanath A. (2014). Temporomandibular joint osteoarthritis: diagnosis and long-term conservative management: a topic review. *Journal of Indian Prosthodontist Society*.

[2] Alford J. W., Cole B. J. (2005). Cartilage restoration, part 1: basic science, historical perspective, patient evaluation, and treatment options. *American Journal of Sports Medicine*.

[3] Murphy M. K., MacBarb R. F., Wong M. E., Athanasiou K. A. (2013). Temporomandibular disorders: a review of etiology, clinical management, and tissue engineering strategies. *The International Journal of Oral & Maxillofacial Implants*.

[4] Kalpakci K. N., Kim E. J., Athanasiou K. A. (2011). Assessment of growth factor treatment on fibrochondrocyte and chondrocyte co-cultures for TMJ fibrocartilage engineering. *Acta Biomaterialia*.

[5] Delatte M., Von Den Hoff J. W., Van Rheden R. E. M., Kuijpers-Jagtman A. M. (2004). Primary and secondary cartilages of the neonatal rat: the femoral head and the mandibular condyle. *European Journal of Oral Sciences*.

[6] Detamore M. S., Orfanos J. G., Almarza A. J., French M. M., Wong M. E., Athanasiou K. A. (2005). Quantitative analysis and comparative regional investigation of the extracellular matrix of the porcine temporomandibular joint disc. *Matrix Biology*.

[7] Detamore M. S., Hegde J. N., Wagle R. R., Almarza A. J., Montufar-Solis D., Duke P. J., Athanasiou K. A. (2006). Cell type and distribution in the porcine temporomandibular joint disc. *Journal of Oral and Maxillofacial Surgery*.

[8] Bailey M. M., Wang L., Bode C. J., Mitchell K. E., Detamore M. S. (2007). A comparison of human umbilical cord matrix stem cells and temporomandibular joint condylar chondrocytes for tissue engineering temporomandibular joint condylar cartilage. *Tissue Engineering*.

[9] Connelly J. T., Vanderploeg E. J., Mouw J. K., Wilson C. G., Levenston M. E. (2010). Tensile loading modulates bone marrow stromal cell differentiation and the development of engineered fibrocartilage constructs. *Tissue Engineering A*.

[10] Seo B.-M., Miura M., Gronthos S., Bartold P. M., Batouli S., Brahim J., Young M., Robey P. G., Wang C.-Y., Shi S. (2004). Investigation of multipotent postnatal stem cells from human periodontal ligament. *The Lancet*.

[11] Bartold P. M., Shi S., Gronthos S. (2006). Stem cells and periodontal regeneration. *Periodontology 2000*.

[12] Ivanovski S., Gronthos S., Shi S., Bartold P. M. (2006). Stem cells in the periodontal ligament. *Oral Diseases*.

[13] Ding G., Liu Y., Wang W., Wei F., Liu D., Fan Z., An Y., Zhang C., Wang S. (2010). Allogeneic periodontal ligament stem cell therapy for periodontitis in swine. *Stem Cells*.

[14] Liu Y., Zheng Y., Ding G., Fang D., Zhang C., Bartold P. M., Gronthos S., Shi S., Wang S. (2008). Periodontal ligament stem cell-mediated treatment for periodontitis in miniature swine. *Stem Cells*.

[15] Wu L., Leijten J. C. H., Georgi N., Post J. N., Van Blitterswijk C. A., Karperien M. (2011). Trophic effects of mesenchymal stem cells increase chondrocyte proliferation and matrix formation. *Tissue Engineering A*.

[16] Acharya C., Adesida A., Zajac P., Mumme M., Riesle J., Martin I., Barbero A. (2012). Enhanced chondrocyte proliferation and mesenchymal stromal cells chondrogenesis in coculture pellets mediate improved cartilage formation. *Journal of Cellular Physiology*.

[17] Meretoja V. V., Dahlin R. L., Kasper F. K., Mikos A. G. (2012). Enhanced chondrogenesis in co-cultures with articular chondrocytes and mesenchymal stem cells. *Biomaterials*.

[18] Livak K. J., Schmittgen T. D. (2001). Analysis of relative gene expression data using real-time quantitative PCR and the 2-ΔΔCT method. *Methods*.

[19] Bruder S. P., Fink D. J., Caplan A. I. (1994). Mesenchymal stem cells in bone development, bone repair, and skeletal regeneration therapy. *Journal of Cellular Biochemistry*.

[20] Gao J., Yao J. Q., Caplan A. I. (2007). Stem cells for tissue engineering of articular cartilage. *Proceedings of the Institution of Mechanical Engineers H: Journal of Engineering in Medicine*.

[21] Tuan R. S., Boland G., Tuli R. (2003). Adult mesenchymal stem cells and cell-based tissue engineering. *Arthritis Research and Therapy*.

[22] Caplan A. I., Dennis J. E. (2006). Mesenchymal stem cells as trophic mediators. *Journal of Cellular Biochemistry*.

[23] Vadalà G., Studer R. K., Sowa G., Spiezia F., Iucu C., Denaro V., Gilbertson L. G., Kang J. D. (2008). Coculture of bone marrow mesenchymal stem cells and nucleus pulposus cells modulate gene expression profile without cell fusion. *Spine*.

[24] Hwang N. S., Varghese S., Puleo C., Zhang Z., Elisseeff J. (2007). Morphogenetic signals from chondrocytes promote chondrogenic and osteogenic differentiation of mesenchymal stem cells. *Journal of Cellular Physiology*.

[25] Wu L., Prins H.-J., Helder M. N., Van Blitterswijk C. A., Karperien M. (2012). Trophic effects of mesenchymal stem cells in chondrocyte Co-cultures are independent of culture conditions and cell sources. *Tissue Engineering A*.

[26] Hubka K. M., Dahlin R. L., Kasper F. K., Mikos A. G. (2014). Enhancing chondrogenic phenotype for cartilage tissue engineering: monoculture and co-culture of articular chondrocytes and mesenchymal stem cells. *Tissue Engineering Part B: Reviews*.

[27] Chen L., Xu Y., Zhao J., Zhang Z., Yang R., Xie J., Liu X., Qi S. (2014). Conditioned medium from hypoxic bone marrow-derived mesenchymal stem cells enhances wound healing in mice. *PLoS ONE*.

[28] Walter M. N. M., Wright K. T., Fuller H. R., MacNeil S., Johnson W. E. B. (2010). Mesenchymal stem cell-conditioned medium accelerates skin wound healing: an in vitro study of fibroblast and keratinocyte scratch assays. *Experimental Cell Research*.

[29] Crigler L., Robey R. C., Asawachaicharn A., Gaupp D., Phinney D. G. (2006). Human mesenchymal stem cell subpopulations express a variety of neuro-regulatory molecules and promote neuronal cell survival and neuritogenesis. *Experimental Neurology*.

[30] di Bernardo J., Maiden M. M., Jiang G., Hershenson M. B., Kunisaki S. M. (2014). Paracrine regulation of fetal lung morphogenesis using human placenta-derived mesenchymal stromal cells. *Journal of Surgical Research*.

[31] Wu L., Leijten J., Van Blitterswijk C. A., Karperien M. (2013). Fibroblast growth factor-1 is a mesenchymal stromal cell-secreted factor stimulating proliferation of osteoarthritic chondrocytes in co-culture. *Stem Cells and Development*.

[32] Makris E. A., MacBarb R. F., Paschos N. K., Hu J. C., Athanasiou K. A. (2014). Combined use of chondroitinase-ABC, TGF-*β*1, and collagen crosslinking agent lysyl oxidase to engineer functional neotissues for fibrocartilage repair. *Biomaterials*.

[33] Gurkan U. A., El Assal R., Yildiz S. E., Sung Y., Trachtenberg A. J., Kuo W. P., Demirci U. (2014). Engineering anisotropic biomimetic fibrocartilage microenvironment by bioprinting mesenchymal stem cells in nanoliter gel droplets. *Molecular Pharmaceutics*.

[34] Spina J., Warnock J., Duesterdieck-Zellmer K., Baltzer W., Ott J., Bay B. (2014). Comparison of growth factor treatments on the fibrochondrogenic potential of canine fibroblast-like synoviocytes for meniscal tissue engineering. *Veterinary Surgery*.

[35] Warnock J. J., Fox D. B., Stoker A. M., Beatty M., Cockrell M., Janicek J. C., Cook J. L. (2014). Culture of equine fibroblast-like synoviocytes on synthetic tissue scaffolds towards meniscal tissue engineering: a preliminary cell-seeding study. *PeerJ*.

[36] Ellman M. B., An H. S., Muddasani P., Im H.-J. (2008). Biological impact of the fibroblast growth factor family on articular cartilage and intervertebral disc homeostasis. *Gene*.

[37] Ellman M. B., Yan D., Ahmadinia K., Chen D., An H. S., Im H. J. (2013). Fibroblast growth factor control of cartilage homeostasis. *Journal of Cellular Biochemistry*.

